# 5G Beyond 3GPP Release 15 for Connected Automated Mobility in Cross-Border Contexts

**DOI:** 10.3390/s20226622

**Published:** 2020-11-19

**Authors:** Gorka Velez, Ángel Martín, Giancarlo Pastor, Edward Mutafungwa

**Affiliations:** 1 Vicomtech Foundation, Basque Research and Technology Alliance (BRTA), Mikeletegi 57, 20009 Donostia-San Sebastián, Spain; amartin@vicomtech.org; 2 Department of Communications & Networking, Aalto University School of Electrical Engineering, P.O. Box 15600, FI-02150 Aalto, Finland; giancarlo.pastor@aalto.fi (G.P.); edward.mutafungwa@aalto.fi (E.M.)

**Keywords:** 5G, connected and automated mobility, V2X, multi-PLMN, cross-border, roaming

## Abstract

Fifth-generation (5G) mobile networks aim to be qualified as the core connectivity infrastructures to address connected automated mobility (CAM), both from a technological and from a business perspective, for the higher automation levels defined by the automotive industry. Specifically, in some territories such as the European Union the cross-border corridors have relevance, as they are the cohesive paths for terrestrial transport. Therefore, 5G for CAM applications is planned to be deployed there first. However, cross-border contexts imply paramount communication challenges, such as seamless roaming, not addressed by current technology. This paper identifies relevant future 5G enhancements, specifically those specified by Third-Generation Partnership Project (3GPP) releases beyond Release 15, and outlines how they will support the ambitions of highly automated driving in cross-border corridors. In order to conduct this study, a set of representative use cases and the related communication requirements were identified. Then, for each use case, the most relevant 5G features were proposed. Some open issues are described at the end.

## 1. Introduction

Automation Levels 4 and 5 (L4 and L5), defined by Society of Automotive Engineers (SAE), will not require someone to take over driving. Such automation levels are expected to be first introduced in highways, as the technical complexity on those roads is lower and the investment profitability prospects are higher compared with urban roads. Cross-border corridors are particularly interesting, as they fit in the highway road type, and they constitute an essential backbone for road transport between countries. However, achieving seamless connectivity that satisfies the needs of connected and automated mobility (CAM) is very challenging in cross-border contexts. The main challenges include roaming and handover latencies, uninterrupted access to required communications and computing resources, data interoperability and accurate geo-positioning.

The vehicle-to-everything (V2X) communication systems can integrate different communication technologies, including cellular systems in centimetre wave (cm-wave) and millimetre wave (mmWave), dedicated short range spectrum (DSRC) and vehicular visible light communications (VVLC). Currently, the automotive industry is putting its effort into cellular systems and DSRC implementations [1]. The 3rd Generation Partnership Project (3GPP) defined the first set of cellular standards for V2X communication under the name long-term evolution (LTE)-V2X in 3GPP Release 14 (Rel-14). LTE-V2X is evolving to 5G, providing the main radio interface to support cellular V2X communication [2]. On the other hand, DSRC vehicular communication technology uses Wi-Fi based physical layer (PHY) and medium access control (MAC) layer protocols following the IEEE 802.11p standard. The adaptation of the widely-used IEEE 802.11 standard for Wi-Fi is being implemented in Europe under the name ITS-G5. As a readily-available, internationally-recognised standard which does not require licensed spectrum, it seems that DSRC technology is well placed. Moreover, some experimental works have already demonstrated its suitability for some specific autonomous driving operations [3]. However, it has a shorter range (few 100 metres), lower data rate, worse spectrum efficiency and more limited quality of service (QoS) management capabilities than 5G [4]. Regardless of the adoption of 5G for CAM, its deployment for consumer electronics is going to be done in any case, fostering new business opportunities for many actors at different levels and sectors [5,6,7], including small and medium-sized enterprises (SMEs) working on data exploitation at cloud level, which is another big incentive for adopting 5G in CAM instead or in combination with DSRC.

There are already some works studying the features to be added and the potential of 6G [8,9]; however, the application of 5G technology in different verticals is still in the experimental stage and needs more research. In 2017, the European Union agreed to establish 5G cross-border corridors with the ambition of having the biggest experimental area in 5G technology. The European Commission, coordinated with public and private actors, is currently focused on these corridors to run CAM projects and to devise digital policies and regulations. Stemming from the work done in one of these ongoing projects, 5G-MOBIX [10], the present article aims to shed some light on the roadmap for deploying SAE L4 and L5 CAM in cross-border roads employing 5G technologies. More specifically, the contributions in this article can be summarised as follows:

- We review the 5G enhancements beyond 3GPP Release 15 (Rel-15) that are most relevant for CAM contexts;
- We identify a set of CAM use cases that target the CAM challenges in cross-border contexts;
- We identify the main communication requirements of the use cases and justify the need for features beyond 3GPP Rel-15 to implement them;
- We propose an implementation, matching the identified use cases and the relevance of 5G features beyond Rel-15, to realise the use cases while satisfying their performance expectations;
- We identify some relevant open issues.

Previous works in the literature do not address the challenges for highly automated driving in cross-border contexts and the new features planned for future 3GPP releases and their relevance there. References [4,6] gave first glimpses of the possibilities of 5G technologies for connected cars, but only addressed up to 3GPP Rel-15. Reference [2] discussed some of the recent technological advances and standardisation developments in cellular vehicle-to-everything (C-V2X) communications, but they did not match them to specific use cases. Reference [1] reviewed the current standardisation efforts focusing on MAC and PHY layers and not on vertical applications. Reference [11] described the general 3GPP Release 16 (Rel-16) features and provided an outlook towards Release 17 (Rel-17) and beyond, but without discussing their applications in vehicular communications and their impacts on autonomous driving. Therefore, the main novelty of this paper is its showing the limitations of the currently available technologies and the possible solutions exploiting planned 3GPP Releases in the context of CAM in cross-border corridors. The insights presented here are valuable not only for the European Union, but also for any 5G cross-border deployment around the world.

## 2. 5G Technologies beyond Release 15

C-V2X has its foundation in the enhancement applied to LTE, as specified in 3GPP Rel-14, to support vehicle connectivity for mostly safety related use cases. This involves use of both short-range direct connectivity and long-range connectivity via existing mobile network infrastructure. Network communications operate over licensed spectrum and messages are transmitted to user equipment (UE) via Evolved Node B (enodeB) using the Uu interface. In contrast, direct communications allow vehicles to directly exchange information using the PC5 interface [1]. 3GPP Rel-15 introduced a set of enhancements for Rel-14 LTE-V2X PC5 and 5G New Radio (NR) technology for the Uu interface. The use of 5G NR leveraged the benefits of increased capacity, reduced latency and improved reliability enabling the environment for more advanced use cases in autonomous mobility that typically require timely and reliable transfers of vast amount of data. However, 3GPP Rel-15 is only an introductory point for 5G NR in C-V2X; specifications of further enhancements are ongoing or were almost complete in subsequent releases. In this section, we highlight some of the new or continued enhancements in 3GPP releases that further strengthen 5G NR’s robustness as a C-V2X solution for advanced automotive use cases in various contexts, including cross-border scenarios.

### 2.1. Roaming

Control plane steering of roaming (CP-SOR) was introduced in Rel-15. This Rrlease supports inter-operator security to deal with the security issues in the inter-operator interface. Rel-16 will include increased home control for authentication and steering of roaming [11]. It will also manage roaming of home routed traffic and local breakout traffic in the visited PLMN. Further enhancements for the 5G CP-SOR are expected in Rel-17, such as the consideration of disaster conditions caused, for instance, by natural hazards. If the available public land mobile networks (PLMNs) indicate that the disaster condition applies, and even if these PLMNs are in the “forbidden PLMN” data field in the SIM, these PLMNs must be considered allowable for registration to the UE while the disaster condition is active [12].

### 2.2. Multi-Radio Dual Connectivity

The road infrastructure may provide connectivity coverage by means of different radio access technologies (RATs), including LTE and 5G NR, operated in different bands and provided by a common operator. Heterogeneous V2X communications can help addressing the bandwidth and scalability requirements that future vehicular networks will face [13]. This may provide an opportunity for a vehicle to utilise simultaneous connections for increased capacity through bonding or enhance reliability through the inherent redundancy. To that end, multi-radio dual (MR-DC) schemes, initially specified in the final phase (“late drop”) of Rel-15, include LTE-NR dual connectivity and NR-NR dual connectivity. In Rel-16, asynchronous NR-NR dual connectivity is specified to avoid the need to collocate gNodeBs (gNBs), providing dual connectivity and reduced dual connectivity setup delays [14]. Furthermore, the space industry is joining in to develop a stack configuration which inter-operates within a 5G architecture [15,16].

### 2.3. Multi-Sim Support

Vehicles traverse areas covered by multiple public land mobile networks (PLMNs) and transit between two PLMN coverage areas (e.g., cross-border). In the multi-PLMN scenario, the vehicle’s home PLMN (original serving network) may have areas with poor QoS or ineffective coverage due to overloading, network failure, etc. In order to ensure vehicle-to-network (V2N) connectivity, the ability of the vehicle to seamlessly switch to or simultaneously utilise a visited PLMN enables safer operation of the vehicle regardless of instantaneous network conditions. The vehicle connectivity to multiple PLMNs would typically require onboard units (OBUs) with several subscriber identity modules (SIMs), one for attaching to each PLMN. Devices ready to host multiple SIMs (multi-SIM) are widely available in the market, mostly using proprietary solutions through device vendors without any support from 3GPP specifications. However, 3GPP has approved a working item in Rel-17 to provide standardised support for multi-SIM UE [17], which can prove beneficial from a reliability perspective.

### 2.4. Nr Positioning

Urban areas with tall buildings or regions with dense foliage require cm-precision positioning. mmWave technology can be exploited to complement global navigation satellite systems (GNSS), and to accurately measure vehicle position [18]. 3GPP Rel-16 introduces native support for positioning on the NR carrier, applicable for mmWave frequencies [19].

### 2.5. Enhanced Sidelink

The short range connectivity between vehicles and roadside infrastructure, pedestrians or other vehicles was originally based on LTE-based sidelink. The use of PC5 interface in LTE sidelinks for C-V2X was specified in both Rel-14 and Rel-15, with limitations associated with legacy LTE technologies. A very detailed overview of sidelink under Rel-14 can be found in [20]. The limitations of Rel-14 satisfying the QoS requirements of several crash scenarios are described in [21]. In Rel-16, a 5G NR sidelink was introduced as a complement to LTE-based sidelink [22]. This brings the higher reliability and low latency benefits of NR to the sidelines, and provides flexibility in spectrum usage for the sidelink by using a dedicated NR spectrum or dynamic sharing of the LTE-Uu or NR-Uu spectrum.

### 2.6. Broadcast and Multicast

Under some conditions and road events, traffic may require urgent alerting in multiple destinations within a local area and for a specific time. To facilitate this delivery model and serve local messages in an efficient manner, broadcast and multicast communication services can make the difference. These communication models might contribute to public and vehicular safety, thereby enhancing operational scalability [23]. Rel-14 included only broadcast without feedback, while Rel-16 will include feedback for increased reliability [24].

### 2.7. Mmwave Communications

The mmWave bands are already identified as enablers for network capacity and throughput enhancements for 5G V2X [25,26]. From the 3GPP perspective, in Rel-15 and Rel-16 the frequency range 2 (FR2: 24,250–52,600 MHz) mmWave bands were specified for 5G NR to complement frequency range 1 (FR1: 410–7125 MHz) bands, opening up new spectrum resources for demanding vertical applications, such as CAM. While FR2 provides at least an order of magnitude higher bandwidth compared to FR1, in Rel-17 there are already efforts being made to evaluate bands from the 52.6–71 GHz region with potentially larger spectrum bandwidths relative to FR2 [27].

As future vehicular systems will demand Gbps links to upload the collected sensor data for advanced automated driving, mmWave bands have become the candidate for 5G spectrum scalability.

### 2.8. Network Slicing

A vehicle may typically support multiple service/traffic types (safety-related and infotainment) associated with different uses cases where each one demands different levels of throughput, latency, reliability, robustness and so on. The different services may mapped to different standardised slice types optimised for the needs of each service. To that end, 3GPP Rel-15 specified three slice/service types (SST) customised for enhanced mobile broadband (eMBB), ultra reliable low latency communications (URLLC) and massive IoT (MIoT) services [28]. The SST slices can be controlled by slice differentiator (SD) values for more granular slice definitions. In Rel-16, roaming support for mutually exclusive access slicing is planned [29]. Thus, slicing will improve roaming performance.

### 2.9. Edge Computing

Advanced vehicular applications and services require ultra-low latency and high bandwidth computing. To alleviate core networks from these demanding requirements and efficiently serve local queries, multi-access edge computing (MEC) technologies bring computing capabilities at the edge of the network [30,31]. Under this connect-compute environment, MEC allows telecommunication operators to flexibly deploy and enhance the computing scalability of networks. Rel-16 provides further enhancements to improve the coordination of mobility procedures with the application. Newer releases will introduce further enhancements to support edge computing for V2X [11].

The value added by each of the previously described 5G features is summarised in Table 1.

## 3. Challenging Cross-Border Use Cases

### 3.1. Identification of Use Cases

As noted in the introduction, cross-border contexts create challenging situations for CAM, specially regarding service continuity. In order to plan the infrastructure and technology required for operating 5G-enabled CAM in cross-border roads it is necessary to conduct some experiments. It is a common approach in engineering to define a set of use cases that represent the most significant interactions with the system under test. 3GPP identified a set of V2X scenarios grouped into five categories [32]: advanced driving, vehicle platooning, extended sensors, remote driving and vehicle QoS support.

Vehicle platooning aims at green driving by grouping a set of vehicles to travel together one after the other. The vehicles that are part of the platoon exchange periodic data to move in a cooperative way. Here, autonomous vehicles can automatically join and leave platoons. Autonomous platooning is expected to be adopted first by trucks, which are some of the main users of cross-border corridors. Platooning optimises transport by using roads more effectively, reducing traffic jams and consequently delivering goods faster.

“Extended sensors” is focused on extending the perception obtained by the onboard sensors, with sensor data received from surrounding vehicles or road side units (RSUs). This way, vehicles generate an enhanced perception of the environment beyond what their own sensors can detect. High-resolution data streams produced by cameras and Lidars impose highly demanding communication requirements. Extended Sensors may require querying data from distant RSUs or vehicles. This could imply communicating with devices located in different countries in a cross-border scenario.

Remote driving enables a remote driver or a V2X application to operate a remote vehicle. In this CAM use case, a remote operator takes control of the vehicle when a breakdown or complex environment impedes the autonomous vehicle’s trajectory. The remote operator can be located in a different country from the vehicle position, so the constraints on a cross-border context may present operational limitations.

Advanced driving implies complex manoeuvres such as overtaking or cooperative collision avoidance that require sharing the driving intentions with the vehicles in proximity. Poor performance of the communication pipeline in terms of latency or reliability may lead to a decrease in the level of automation and/or increase likelihood of accidents. Such a safety-critical use case needs to be tested in as many situations as possible, including roaming or supporting MECs when located in different countries.

Vehicle QoS support could be considered a horizontal use case, whereby the vehicle is able to anticipate network QoS fluctuations and adjusts its service requirements accordingly. Moreover, the network is able to allocate connectivity resources to preserve a QoS to satisfy application needs and the application can adapt its traffic demands to meet network performance. The overall goal is to offer a smooth user experience, meaning the coordination of different network infrastructures of both sides in a cross-border context to perform seamless transitions.

### 3.2. Use Cases’ Requirements

Each of the identified use cases can be implemented with different levels of automation while they all require high performance parameters in terms of connectivity. In this paper we focus on highly automated driving, that is, SAE L4 and L5. Here, the requirements are even more demanding as there is no handover to manual driving.

**Figure 1:**
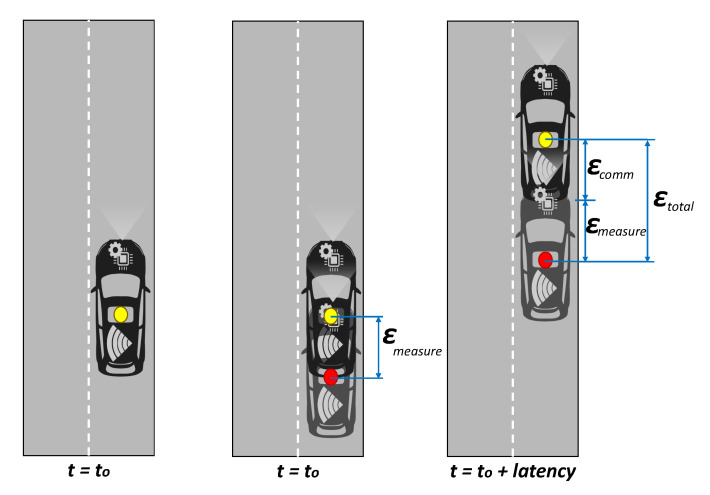
Left to right in the figure: (1) Actual position of the vehicle when the message is sent ($t=t_{0}$). (2) Position estimated by the onboard positioning system. This is the position communicated. Note that there is a $\varepsilon_{measure}$ longitudinal error. (3) Actual position of the vehicle at $t=t_{0}+latency$. This is the instant in time that the message is received by another vehicle. There is a $\varepsilon_{comm}$ error caused by the network latency. The receiver needs to deal with a $\varepsilon_{total}$ offset.

The spatial- and time-accuracy required by L4 and L5 autonomous vehicles with respect to object localisation are key factors when translating the nominal performance of communication technologies to operational parameters of use cases. The latency in the positioning messages received from another vehicle or entity adds uncertainty to the transmitted localisation value, with a higher impact on the longitudinal direction. This effect is depicted in Figure 1 for the longitudinal error.

The localisation requirements for autonomous vehicles were studied in [33], and they concluded that a maximum lateral error of 0.57 m and a maximum longitudinal error of 1.40 m are acceptable for passenger vehicles travelling on highways—the most common road type at cross-border corridors. Considering that state-of-the art ego-vehicle localisation methods have in general sub-metre accuracy in the order of several decimetres [34], there is little room for positioning error induced by network latency. To study the potential effect of the latency on the localisation error, we have measured the position error induced by different latency values using a public driving dataset. More specifically, we have used three highway scenarios from the CommonRoad dataset [35]. The scenarios were partly recorded from real traffic and partly hand-crafted to create challenging situations. The selected scenarios include lane merges and curves to make them more challenging. More information about the selected CommonRoad dataset scenarios can be found in Table 2.

The dataset provides position, velocity, acceleration and heading values of each vehicle recorded at 10 Hz, which is the standard frequency for sending this kind of vehicle data [36]. In this study and based on SAE J2945/1 [37], the receiver estimates the current position of the transmitter vehicle based on the latest received message assuming that the transmitter vehicle is moving at a constant acceleration and heading. For each vehicle position data in the dataset, we have estimated the position after a certain time lapse that would correspond to the message latency. The position error added by the latency is calculated as the Euclidean distance between the vehicle position estimation and the actual position at the time that the message reaches the receiver. This ground truth is obtained by using a quadratic interpolation between the closest points that are part of the dataset.

**Figure 2:**
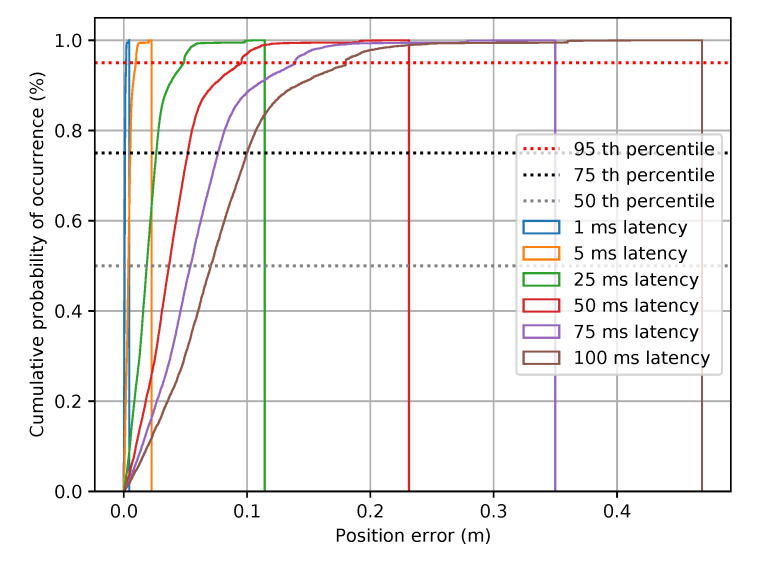
Cumulative distribution function of the positioning error added by the latency in a highway scenario.

As specified by the European Committee for Standardization (CEN) and European Committee for Electrotechnical Standardization (CENELEC) [38], positioning accuracy is represented with a set of three statistical values given by the 50th, 75th and 95th percentiles of the cumulative distribution function (CDF) of the position error. The empirical CDF obtained in our study with the datasets of Table 2 is depicted in Figure 2 for latencies in the range of 1 to 100 ms. A latency higher than 100 ms would make impossible the real-time processing of the received data, this being the maximum tolerable latency for vehicle-to-vehicle (V2V) communication [39]. For a latency of 25 ms or below, the error’s 95th percentile is below 5 cm, which is negligible for a localisation problem. With 50 ms latency, the 95th percentile is almost 10 cm, and reaches 18 cm with 100 ms latency. Depending on how close the transmitter vehicle’s positioning measurement errors are to the error bounds, these decimetre level errors added by the network latency can definitely affect to the localisation of the transmitter vehicle in the receiver vehicle’s frame. It is also important to consider that even if the network latency does not increase the localisation error outside of the error bounds, the positioning message needs to be received with enough time in advance to trigger a timely reaction.

3GPP defined some performance requirements in [32] for use cases involving highly automated driving that are supposed to consider all these aspects. The performance requirements are summarised in Table 3. Note that the latency requirements are in line with previous calculations; thus, a latency lower than 50 ms is required by all use cases, and it is much lower for advanced driving and remote driving due to their safety-critical nature and for extended sensors because of the required synchronisation and alignment of perception data coming from different sensors (e.g., video stitching or Lidar point cloud fusion). Extended sensors stands out as the most demanding use case in terms of data rate and communication range. Previous 5G-V2X requirements compiled by 3GPP noted modest data rate requirements for remote driving under the assumption that a vehicle only transmits lightweight processed data [40]. However, more advanced remote driving approaches involve transmission of raw sensor data, so the whole onboard sensor suite can be processed in a remote server. This would of course require some data rate requirements similar to or even more demanding than extended sensors. Advanced driving requires a high transmission rate of 100 messages per second. This is exactly the same recommendation given in [33], derived as the time required between successive localisation updates. Vehicle platooning does not stand out in any of the performance indicators proposed by 3GPP, but the requirements as a whole are still very demanding.

The latency of V2V communication under a LTE network for multi-operator environments with regional split was studied in [41] and a latency of 58 ms was estimated in inter-operator communications without inter-operator handover. In the same work, the inter-operator handover, when vehicles have to detach from one operator and then attach to the other one, was estimated to have 300 ms of latency. It is then clear than enhanced 5G features are required to meet the demanding latency requirements.

To sum up, the identified CAM use cases have demanding performance requirements that cannot be met by LTE infrastructures. This is even more clear in a cross-border context that requires roaming. The following section proposes some implementation options to overcome this issue that exploit features present at 3GPP Release 16 and beyond.

## 4. Cross-Border Use Case Implementations

The different communication components coming from Section 2 are essential enablers for the use cases described in Section 3, as they play specific roles in the communications pipeline, as described hereafter. Therefore, the Table 4 summarises the relevance of the different communication technologies to meet the requirements of the categorised use cases.

The implementation and deployment aspects of the different 5G features are summarised in Table 5. It includes some overheads to take into account in the development of the CAM service and investment efforts on infrastructures and systems of operators aiming to ease and catalyse the operation of innovative CAM applications in cross-border contexts with multi-domain networks.

**Figure 3:**
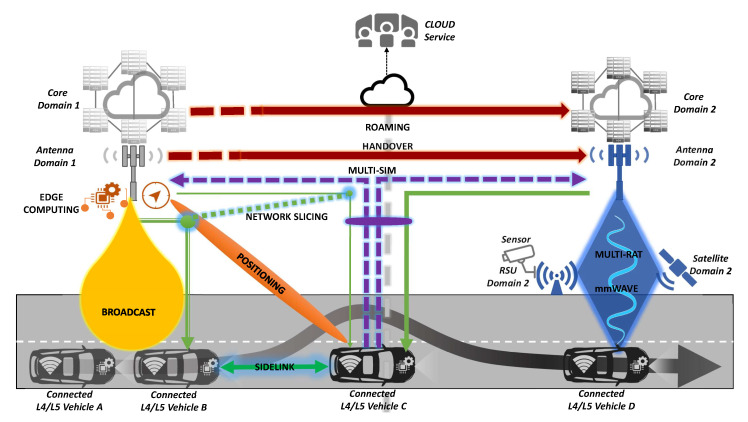
General scenario of the proposed solution.

In the Figure 3, all the 5G communication technologies come into place for the previously identified use cases, where cross-border context brings further complexity when systems operated by different operators participate.

Specifically, multiple radio technologies expand cellular connectivity and allow the utilisation of the most appropriate option depending on coverage context. Here, mmWave communications are gaining prominence because of their possibilities for infrastructure checkpoints, suitable for public infrastructures, accumulating traffic and serving/consuming spontaneous traffic floods using mmWave bandwidths. Furthermore, satellite communications are intended to augment coverage extension and resilience when managing redundancy on terrestrial and satellite paths. For critical communications, where redundancy could be a must, the duplication of the traffic to be delivered through two available networks enforces the reliability of communication and the exploration of different paths which could get lower latency. Particularly, in the automotive field, the positioning is a key feature where NR advances can improve the accuracy and compensate for the limitations of widely employed geo-position sensors shipped by the connected cars. Then, NR sidelink facilitates opportunistic communication with entities in the surrounding area. It is essential from a business perspective as it enables decentralised communication in areas where ad hoc infrastructure is not available yet. Furthermore, the deployment of advanced techniques which boost handover and roaming is imperative to keeping latency low and realising the communication with external systems, as they are onboard while travelling. In regard to applications or services wherein the audience volume is big, common information awareness is required immediately and explicit acknowledge messaging is not suitable, broadcast and multicast communication turns into a primary option. Widely studied techniques to accommodate traffic demands to network assets and to prioritise specific flows can make the difference when traffic with different severity levels is delivered. Here the harmonisation of different operator policies is a complex challenge. Last but not least, capillarity, zero latency and local privacy from edge computing systems will bring environmental understanding to the next level comprising real-time computer vision and network analysis as a perfect example of incoming service and network symbiosis.

### 4.1. Advanced Driving

**Figure 4:**
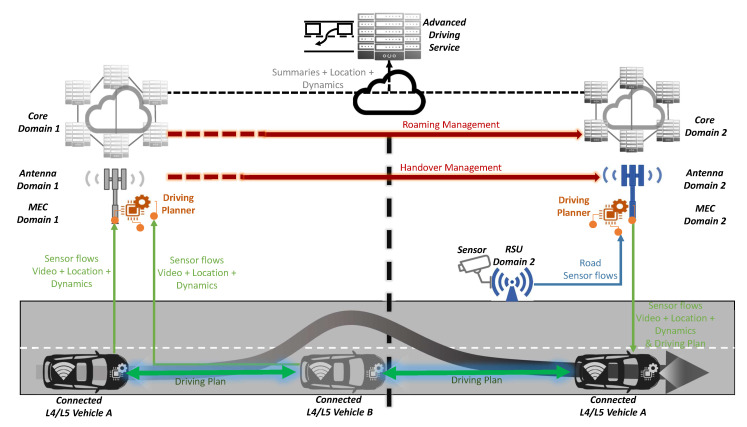
Advanced driving scenario of the proposed solution.

The advanced driving use case involves the participation of different vehicles and infrastructures in order to cooperate and provide information for planning and operating a complex manoeuvre, as depicted in Figure 4. Here, each participant may use a different communication technology. Thus, the interoperability provided by multi-modal/multi-radio technologies of 5G [14] is critical to allow the information exchange from all the surrounding actors. Moreover, sidelink communications such as PC5 C-V2X and 5G NR sidelink [22] add flexibility and versatility enabling data flows between peers once the parties are connected and roles are accepted. As the manoeuvre requires a synchronised response and coordination from vehicles, the network needs to deal with the timely delivery of messages to foster a solid and consistent knowledge at once. Here, the generalied Precision Time Protocol (gPTP) provides reliable time synchronisation as declared in 3GPP TS 23.501 [28]. Furthermore, as the scenario timeframe for such advanced driving use cases is usually small, it is important to minimise communications interruptions and outages while some participants are migrating towards other cells (handover) or other networks (roaming) with low-latency migrations between the network infrastructures.

From the latency perspective, advanced roaming techniques [11] boost the migration of sessions along vehicles, and sidelink communications [22] simplify the protocol to exchange coordination messages to create more direct and quick communication among the surrounding participants. Specifically, roaming techniques get more complex in cross-borders to speed up data sessions across different systems and domains.

Accurate positioning from 5G NR [19] with meter accuracy further enhanced with the next 3GPP releases will bring sub-meter accuracy, and will complement onboard sensor-based measures; multicast networking bridged by FeMBMS (further evolved multimedia broadcast multicast service) technology [24] will ease synchronous data sharing across the manoeuvring participants; and edge computing will provide higher-level vision of the driving situation by gathering data from all the actors in place. Overall, such additional features, could enforce the use case results, adding data accuracy, boosting communications protocols and reducing exchanged messages, thereby gaining efficiency from reduced overheads.

This use case also includes RSUs, V2X communications and multi-PLMN aspects as declared in 3GPP TR 22.886 [40], but it can go beyond by creating of a multi-tier architecture for the server adding edge computing services [11] to provide the advanced driving service with close-to-zero latency and generating summaries and reports for a central server monitoring.

### 4.2. Vehicle Platooning

**Figure 5:**
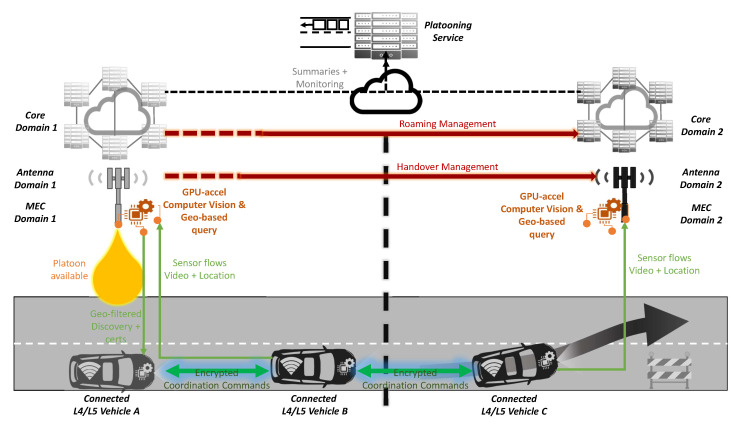
Platooning scenario of the proposed solution.

The vehicle platooning use case has some similarities with advance driving, but here participants are not just exchanging information to facilitate a safe manoeuvring, as shown in Figure 5. Instead, some participants with a common path enrol in a platoon and receive instructions from the leading vehicle. This use case brings a big challenge in the cross-border domain, as it involves a higher number of actors which will move across different borders and networks. Here, the synchronised acknowledgement among the platoon vehicles and the leader is mandatory, where the gPTP protocol is key to providing reliable time synchronisation as declared in 3GPP TS 23.501 [28], granting a common time base for all participants. This becomes more complex as the membership dynamics need to be managed by the platoon, for example, when a vehicle joins or leaves the platoon, requiring the re-arrangement of the vehicles. In order to ease the communication between the platoon vehicles and to keep the connection alive, the sidelink communications, such as PC5 C-V2X and 5G NR sidelink [22], play a significant role. Furthermore, the multicast communications minimise the potential inconsistencies for a common awareness distribution when sharing information along the platoon. Here, FeMBMS technology and the advances to include feedback in the next releases [24] will facilitate information sharing and actuation coordination. The multicast communications would also simplify the platooning protocol, reducing overheads. Last, the mid/long distance covered makes this use case more prone to failures, that is, communications interruptions when performing cell handover or cross-border roaming, requiring low-latency and reliable performance.

From the latency perspective, advanced roaming techniques [11] and sidelink communications [22] make again the difference. Furthermore, broadcast and multicast [24] help to simplify coordination messaging by removing acknowledgement and clock messaging for common shared information while granting distributed awareness at once. Ensuring consistent timing and synchronous distribution of messages [28] for broadcasting gets intricate in cross-borders where different systems operate by different operators with shifted time zones get place.

Additionally, the same benefits from extra features apply. Accurate positioning of 5G NR [19] complements onboard sensor-based measures; edge computing [11] provides a higher level vision of the driving situation, gathering data from all the members in place and the QoS management of the traffic from a platoon when managing co-located services. The network slicing techniques [29] provisioning virtual/logical network assets devoted to specific traffic and isolating the performance from concurrent traffic flows could reinforce the use case results, adding data accuracy while enabling cost–performance trade-offs.

This use case embraces RSUs, V2V and broadcast/multi-cast communications as proposed in [40]. Furthermore, part of the processing load and the clustering of information to the platoon’s region of interest can be managed by the edge computing infrastructures to gain scalability.

### 4.3. Extended Sensors

**Figure 6:**
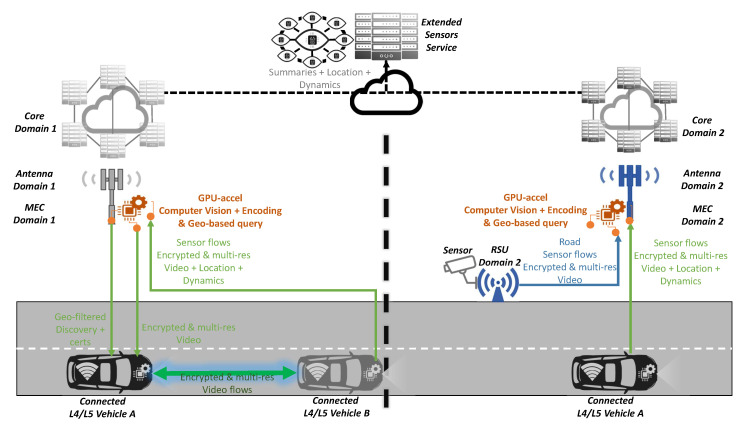
Extended sensors scenario of the proposed solution.

“Extended sensors” aims to expand data and environmental knowledge with information coming from sensors from surrounding systems such as RSUs or vehicles, as illustrated in Figure 6. The heterogeneity on the network interfaces of different systems makes multi-radio [14] support necessary. Once the discovery protocol has been performed and the handshake between the data source and consumer is done, the sidelink communications, such as PC5 C-V2X and 5G NR sidelink [22], allow peers to communicate without management overheads from the network infrastructure. Here, specifically the edge computing architecture [11] makes the difference, as the overheads for onboard computing resources can be offloaded to edge processing resources compiling, filtering and processing the raw data from vehicles to provide onboard systems with trusted and relevant metadata to be used by the onboard driving systems.

From the latency perspective, sidelink communications [22] make participant communications more direct and quicker. Furthermore, zero latency of edge computing enabled services [11] will bring environmental understanding closer to the users empowering systems shipped at the vehicles with more additional sensor sources. Specifically, edge services linked to discovery and security can boost quality negotiation, selection of region of interest and credentials exchange with an edge entity with a higher standing point to manage configurations according to coordination policies and local performance context, including radio link congestion or vehicles’ computation capacity. In a cross-border context, the utilisation of different edge providers would need common and standard protocol stacks and formats to ensure that common APIs and seamless service provisioning could be done, decoupling services from sensors and infrastructure vendors.

Again, features such as positioning of 5G NR [19], based on the use of a location server similar to LTE, may help to improve accuracy from sensors. This architecture-wise technique employs a positioning reference signal (PRS) for the downlink-positioning, correlating the time of arrival (ToA) and sounding reference signals (SRSs) for the uplink-positioning, estimating the received power, the angle of arrival and the round-trip time (RTT). Moreover, broadcast and multicast communications [40] could ease the discovery protocol of the available sensors, especially as singular video streams from sensors ahead are repeatedly subscribed by vehicles at the back. In any case, here, the speed and reliability of network handover and roaming are not so critical, as the onboard driving systems must be autonomous and the goal is to find and consume potentially relevant data streams from outside the vehicle.

For the QoS slicing feature it is important here to include processing features of the video stream destination to match the data source, not only with the network performance as described in [40], but also with the computation capacity of the vehicle consuming a data flow.

### 4.4. Remote Driving

**Figure 7:**
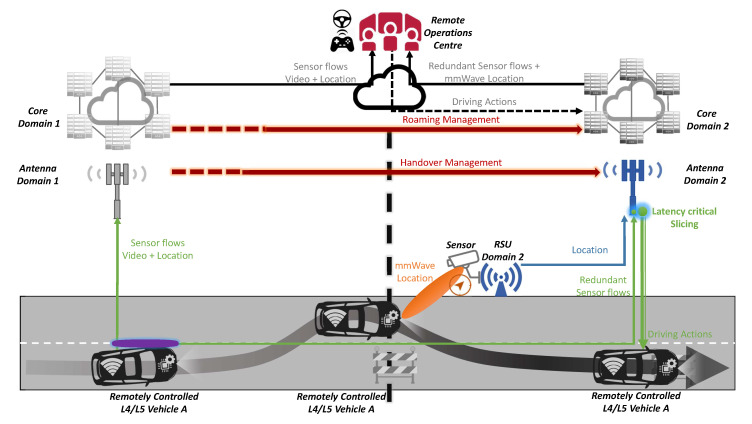
Remote driving scenario of the proposed solution.

In this use case the volume of information sent to the operator is big; the latency of remote actions must be low; the accuracy of positioning is helpful, especially for high speeds; and the reliability and stability of communications are essential to ensure continuous control. To this end, the multi-modal/multi-radio communications [14] are key to operate through an appropriate radio access when the coverage of some of them falls, as presented in Figure 7. Furthermore, the QoS slicing mechanisms [29] can ensure a minimal QoS to allow a coarse-grained environmental view yet a practical one. This use case brings main aspects from mission-critical communications, where the availability of a network slice isolates traffic avoiding bottlenecks or transitory outages potentially introduced by other services or applications. In any case, quick and seamless network handover and roaming are mandatory.

From the latency perspective, the utilisation of multiple radio technologies is crucial to providing substitute connectivity when cellular radio technology violates the latency threshold. This manner, cellular coverage is augmented through alternatives and 5G vision aims to embrace heterogeneous technologies and contexts [14] with a roadmap to merge technologies in public and private networks and bands. In this regard, network management tools to operate network slicing are also essential to ensure that critical operations are prioritised to other traffic [29]. The harmonisation of the provided slice in a multi-domain infrastructure is especially complicated in cross-border contexts, where each operator deploys its own policies and technology stack to monitor and control the network setup.

In this case, sidelink communications are not required as the communication is between the cloud service and the controlled vehicle. The broadcast and multicast communications have no use for this pure unicast data flow. However, in this V2N scenario, a multi-SIM approach would be very valuable [17]. If one PLMN fails, the other one can be used in order to ensure the continuity of the remote driving service. In contrast to the rest of use cases, this use case has no V2V or vehicle-to-infrastructure (V2I) element and depends only on V2N, so the whole use case would be disabled in cases wherein the link with the network fails. Edge computing does not play a key role, as no processing tasks need to be offloaded from vehicles or cloud services to the edge.

### 4.5. Vehicle QoS Support

**Figure 8:**
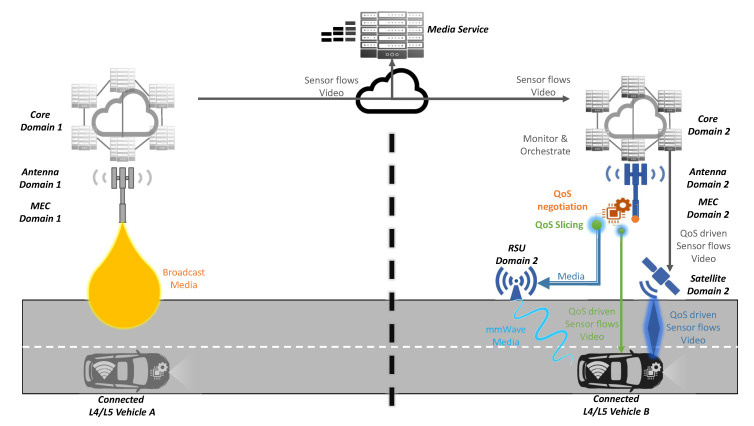
Quality of experience scenario of the proposed solution.

This use case is transverse and intrinsically related to compensate network issues by finding new ways to complement or substitute the under-performing radio link, as rendered in Figure 8. Thus, multi-radio [14] communications, broadcast and multicast [24] flows, mmWave [27] spectrum bands and network analysis at local edge [11] will lead to QoS slicing [29], ensuring a seamless and steady QoS. Even if it is not key, a multi-SIM [17] support would also be interesting to increase the reliability of the service.

From the latency perspective, as this use case is transverse, the manner that multi-radio, broadcast and network slicing technologies may help to reduce the use case latency was already described.

This use case is wider in terms of multi-modal communications, adding satellite communications—for widening coverage—mmWave communications, to increase bandwidths in a short-range area and vehicle traffic tethering, allowing a vehicle to act as a gateway for another vehicle connected through V2V connection.

## 5. Conclusions and Open Issues

In this paper we gave an overview the different 5G features from Release 16 and 17 that will be key to enabling or boosting use cases of the CAM domain, specifically in the context of cross-borders. To this end, we took advanced CAM use cases targeted in 3GPP documents and put them into the cross-border context to identify further communication requirements. Then, we analysed the roles and relevance of identified 5G features to meet CAM requirements, such as: low-latency roaming to ensure service continuity; multi-radio communications to gain interoperability of cross-border networks; multi-SIM connectivity to increase reliability when crossing a border; NR positioning to enhance localisation accuracy and mitigate localisation errors induced by roaming latency; sidelink to further enable distributed architectures; broadcast modes to efficiently share common data; mmWave to expand radio capacity; network slicing to book resources to ensure QoS; and edge computing to deploy a scalable infrastructure. This paper presents a 5G deployment option for CAM over a terrestrial network inspired by the work done in 5G-MOBIX, where other options like satellite communication are also considered.

However, there are some open issues not covered in ongoing discussions and working documents of 3GPP. Firstly, there is a lack of standard MEC APIs to manage the servicing lifecycle, from release and deployment to operation and management. In a cross-border context this would mean extra and manual work to deploy a MEC service on a network and migrating or cloning it to another one. Moreover, a seamless MEC handover mechanism is also lacking. The MEC system should have a standard mechanism to transfer the session data to the following MEC node as the devices physically navigate along them, including MEC nodes located in different PLMNs.

As shown in Section 3, the latency of the V2X messages is critical. In a cross-border context, the roaming adds even more latency and makes the timely delivery of messages to a set of distributed systems very challenging, complicating a common understanding and creating inconsistencies and conflicts. In Ethernet networks, where there is no roaming at all, this is addressed by applying time-sensitive networking standards that ensure bounded latency instead of a best-effort approach. However, wireless networks behave differently from wired links, due to device mobility and the intrinsic characteristics of the physical layer. This paper already proposes some options to minimise latency problems by using 5G technologies such as slicing, edge computing or mmWave communications. Nevertheless, a standard strategy for resource overprovisioning and traffic congestion prediction and management can help mitigate this problem even more.

From the business feasibility perspective, the deployment and exploitation options of 5G in cross-border corridors should be studied further. Several aspects need to be considered for a successful deployment of 5G for CAM, such as the base station and MEC density or the backhaul provision. Cost reduction solutions are also necessary that promote infrastructure sharing, and exploit cooperative models between the automotive industry, mobile network operators, road operators and new players such as cloud or edge service providers.

## Figures and Tables

**Table 1:** Added value brought by 5G technologies to cross-border connected automated mobility (CAM) services.

5G Technology	Reference	Added Value
Roaming	[11]	Service continuity in cross-border contexts.
Multi-Radio	[14]	Increased interoperability by supporting
		multiple RATs of cross-border networks.
Multi-SIM	[17]	Increased reliability while crossing the border
		based on SIM redundancy.
NR positioning	[19]	Increased positioning accuracy, complementing current sensors
		and mitigating the positioning error induced by roaming latency.
NR sidelink	[22]	Nearby device-to-device communication
		without the need of a base station.
Broadcast &	[24]	Improved resource efficiency compared to unicast
multicast		for transmitting common data of interest.
mmWave comm.	[27]	Expanded radio capacity. Gigabit-per-second transmission rates.
Slicing	[29]	Dedicated slice to support the requirements
		of a particular cross-border CAM service.
Edge	[11]	Offloading computing loads to vehicular edge computing nodes
Computing		via vehicle-to-infrastructure links.

**Table 2:** CommonRoad dataset scenarios used to study the effect of latency in the positioning error.

Scenario Name	Scenario Class	Duration (s)	Number of Dynamic Vehicles
CHN_Cho-2_8_T-1	Highway	15.0	26
CHN_Cho-2_10_T-1	Highway	16.5	18
DEU_Stu-1_5_T-1	Highway	16.0	22

**Table 3:** Performance requirements for highly automated use cases according to 3GPP TS 22.186 [32].

			Max e2e			
	Payload	Tx Rate	Latency	Reliability	Data Rate	Comm. Range
Use Case	(Bytes)	(message/sec)	(ms)	(%)	(Mbps)	(m)
Advanced Driving	2000–12,000	100	3–10	99.999	30–53	500
Vehicle Platooning	50–1200	30	10	99.99	50–65	80–350
Extended Sensors	-	-	3–50	99–99.999	10–1000	1000
Remote Driving	-	-	5	99.999	25(UL)/1(DL)	-

**Table 4:** A qualitative assessment of the relevance of communication technology to support use cases in cross-border contexts.

	Advanced	Vehicles	Extended	Remote	QoS
5G Technology	Driving	Platooning	Sensors	Driving	Support
Roaming	✓✓	✓✓	✓	✓✓	✓
Multi-radio comms.	✓✓	–	✓✓	✓✓	✓✓
Multi-SIM	–	–	–	✓✓	✓
NR Positioning	✓	✓	✓	✓✓	–
NR sidelink	✓✓	✓✓	✓✓	–	–
Broadcast & multicast	✓	✓✓	✓	–	✓✓
mmWave comms.	–	–	–	–	✓✓
Network slicing	✓	✓	✓	✓✓	✓✓
Edge computing	✓	✓	✓✓	–	–

✓✓ The technology is key for the use case’s requirements. ✓The technology is relevant for the use case’s requirements. —The technology is not required to support the use case.

**Table 5:** Implementation and deployment aspects of 5G technologies in cross-border CAM services.

5G Technology	Implementation	Deployment
Roaming	Availability of common APIs	Continuity on IP address
Multi-radio	Aggregate traffic	Multiple network interfaces
	Transform across radio technologies	Dedicated infrastructure
Multi-SIM	Perform techniques for payload merge	Accurate clock distribution
	and payload discard based on timestamps	technique granting common time basis
NR positioning	Apply sync to clock and timestamp signalling	Dedicated infrastructure
	Local master/slave clocks	Hierarchical clock sync setup
NR Sidelink	Manage discovery	Edge system to boost discovery
	Negotiate handshake & security	Edge system to enable trust
Broadcast &	Employ compatible UE	Aggregate dedicated system
multicast	Remove dependency on feedback messages	to the Base Stations
mmWave comm.	High-performance bus	High-throughput equipment
	High-capacity storage	High-capacity cache
	Employ compatible UE	Short-range deployment
Slicing	Policies implementation	Policies harmonisation across domains
	Interfacing provisioning/virtualization system	Open 3rd party APIs
Edge	Mobility-based session continuity	Listen neighbour activity updates
Computing	Geo-parcelling for instance subscription	Geo-pinned deployment

## Abbreviations

The following abbreviations are used in this manuscript:

3GPP	Third-Generation Partnership Project
API	Application Programming Interface
C-V2X	Cellular Vehicle-to-Everything
CAM	Connected Automated Mobility
CDF	Cumulative Distribution Function
CEN	European Committee for Standardisation
CENELEC	European Committee for Electrotechnical Standardisation
CP-SOR	Control Plane Steering of Roaming
DL	Downlink
DSRC	Dedicated Short Range Spectrum
eMBB	enhanced Mobile Broadband
FR	Frequency Range
GHz	Gigahertz
gNB	gNodeB
GNSS	Global Navigation Satellite System
GPU	Graphics Processing Unit
IEEE	Institute of Electrical and Electronics Engineers
L4	Level 4
L5	Level 5
LTE	Long Term Evolution
MAC	Medium Access Control
MEC	Multi-access Edge Computing
MHz	Megahertz
MIoT	Massive Internet of Things
mmWave	millimetre Wave
MR-DC	Multi-Radio Dual Connectivity
NR	New Radio
OBU	On-Board Unit
PHY	Physical Layer
PLMN	Public Land Mobile Network
QoS	Quality of Service
RAT	Radio Access Technology
Rel-14	Release 14
Rel-15	Release 15
Rel-16	Release 16
Rel-17	Release 17
RSU	Road Side Unit
SAE	Society of Automotive Engineers
SD	Slice Differentiator
SIM	Subscriber Identity Module
SME	Small and Medium-sized Enterprises
SST	Slice/Service Types
TR	Technical Report
UE	User Equipment
UL	Uplink
URLLC	Ultra Reliable Low Latency Communications
V2I	Vehicle-to-Infrastructure)
V2N	Vehicle-to-Network
V2V	Vehicle-to-Vehicle
V2X	Vehicle-to-Everything
VVLC	Vehicular Visible Light Communication
